# Disruptions to schistosomiasis programmes due to COVID-19: an analysis of potential impact and mitigation strategies

**DOI:** 10.1093/trstmh/traa202

**Published:** 2021-01-29

**Authors:** Klodeta Kura, Diepreye Ayabina, Jaspreet Toor, T Deirdre Hollingsworth, Roy M Anderson

**Affiliations:** London Centre for Neglected Tropical Disease Research, London, UK; Department of Infectious Disease Epidemiology, School of Public Health, Faculty of Medicine, St Mary's Campus, Imperial College London, London, UK; MRC Centre for Global Infectious Disease Analysis; Big Data Institute, Li Ka Shing Centre for Health Information and Discovery, University of Oxford, Oxford OX3 7LF, UK; Big Data Institute, Li Ka Shing Centre for Health Information and Discovery, University of Oxford, Oxford OX3 7LF, UK; Big Data Institute, Li Ka Shing Centre for Health Information and Discovery, University of Oxford, Oxford OX3 7LF, UK; London Centre for Neglected Tropical Disease Research, London, UK; Department of Infectious Disease Epidemiology, School of Public Health, Faculty of Medicine, St Mary's Campus, Imperial College London, London, UK; MRC Centre for Global Infectious Disease Analysis; DeWorm3 Project, Natural History Museum of London, London, UK

**Keywords:** COVID-19, elimination as a public health problem, mass drug administration, modelling, schistosomiasis

## Abstract

**Background:**

The 2030 goal for schistosomiasis is elimination as a public health problem (EPHP), with mass drug administration (MDA) of praziquantel to school-age children (SAC) as a central pillar of the strategy. However, due to coronavirus disease 2019, many mass treatment campaigns for schistosomiasis have been halted, with uncertain implications for the programmes.

**Methods:**

We use mathematical modelling to explore how postponement of MDA and various mitigation strategies affect achievement of the EPHP goal for *Schistosoma mansoni* and *S. haematobium*.

**Results:**

For both *S. mansoni* and *S. haematobium* in moderate- and some high-prevalence settings, the disruption may delay the goal by up to 2 y. In some high-prevalence settings, EPHP is not achievable with current strategies and so the disruption will not impact this. Here, increasing SAC coverage and treating adults can achieve the goal. The impact of MDA disruption and the appropriate mitigation strategy varies according to the baseline prevalence prior to treatment, the burden of infection in adults and the stage of the programme.

**Conclusions:**

Schistosomiasis MDA programmes in medium- and high-prevalence areas should restart as soon as is feasible and mitigation strategies may be required in some settings.

## Introduction

Schistosomiasis is a parasitic disease affecting millions of people in several endemic regions.^1^ Intestinal (caused by *Schistosoma mansoni* or *Schistosoma japonicum*) and urogenital (caused by *Schistosoma haematobium*) are the two most prevalent forms of human schistosomiasis.^2^ At present, mass drug administration (MDA) of praziquantel to school-age children (SAC; 5–14 y of age) is the main method of reducing the burden of morbidity associated with this infection.^3,4^ Control programmes additionally include recommending behaviour modification and improvements in sanitation to lower the intensity of transmission.^5,6^

MDA is mostly targeted at SAC since age-intensity profiles are convex in shape, with a peak in infection levels typically seen among SAC and teenagers.^7,8^ Additionally, this age category can be reached through school-based treatment programmes, which have been shown to be cost-effective in reaching these populations.^9^ It should be noted that in some high-risk areas, treatment of adults is also recommended.^10^

The 2030 World Health Organization (WHO) target for schistosomiasis is elimination as a public health problem (EPHP), achieved when the heavy-intensity prevalence in SAC decreases to ≤1%.^11,12^ For *S. mansoni*, heavy-intensity infection is defined as having ≥400 eggs/g of faeces and for *S. haematobium* is defined as having ≥50 eggs/10 mL of urine.^13^ Heavy-intensity infections can be diagnosed by using the Kato–Katz technique and urine filtration.^14–16^ Morbidity is thought to be associated most strongly with these heavy burdens and hence they are the target to reduce the number of infections.

Previous mathematical modelling for schistosomiasis has shown that EPHP can be achieved in low- (<10% baseline prevalence among SAC) to moderate-transmission (10–50% baseline prevalence among SAC) settings, but in certain high-transmission settings (≥50% baseline prevalence among SAC), inclusion of adults in MDA programmes would be needed to achieve the EPHP goal.^2,11,17–19^

Due to the coronavirus disease 2019 (COVID-19) pandemic, the WHO has advised governments to postpone MDA for schistosomiasis (and other neglected tropical diseases).^20^ It is likely that the MDA postponement will have different impacts in different transmission settings, as the level of resurgence or bounce-back will vary across settings since this depends on the magnitude of the basic reproductive number, R_0_. In particular, we expect the postponement to have a greater impact in high-transmission settings, since resurgence will be faster in these areas due to higher rates of transmission (larger values for R_0_). Although missed MDAs will certainly lead to resurgence in infection levels, the epidemiological impact of such postponement is poorly understood at present, but models of parasite transmission can provide important insights.

The stage (how many rounds of MDA prior to the delay) and effectiveness of the programme (coverage and compliance) will play a role in the resurgence or bounce-back rate.^21^ Programmes in their early stages may return to pretreatment endemicity levels faster, whereas programmes^22^ that have managed to significantly reduce the intensity of transmission will see lower levels of resurgence, provided the transmission rate is not too high. However, in high-transmission settings, programmes in later stages will have a risk of losing much of the long-term benefit of multiple rounds of MDA.

In this article we use a mathematical model of parasite transmission and control by MDA to estimate the impact of temporarily delaying MDA on achieving the EPHP goal. We consider a range of transmission settings and investigate the impact of missing one round of treatment.

## Methods

### Transmission model

We employed an age-structured deterministic model developed by the Imperial College London.^8,23^ The model incorporates treatment by MDA and is parameterised for *S. mansoni* and *S. haematobium* with previously published data and estimated parameter values derived from past epidemiological studies (Supplementary Table S1).^17,24^ Briefly, the model describes the dynamics of the adult worms in the human host population and a single reservoir of infectious material (infected snails are short lived).^23^ This model assumes a negative binomial distribution of parasites per host with a fixed aggregation parameter, k (density-dependent fecundity), and assumes monogamous sexual reproduction among worms. The egg contribution to the reservoir depends on the age-specific contact rate for each individual in the population.

The numerical simulations were run for a single community with a population size set at 1000, assuming no migration. In our simulations, treatment is delivered at random in each round, i.e. no systematic non-adherers and no individuals without access to treatment. Acquired immunity is not taken into consideration.^25^ To simulate moderate and high baseline prevalence settings (for low and high adult burden of infection), the intrinsic intensity of transmission, i.e. R_0_, is varied (higher prevalence settings corresponding to higher R_0_ values).

### Scenarios and mitigation strategies

**Figure 1.**
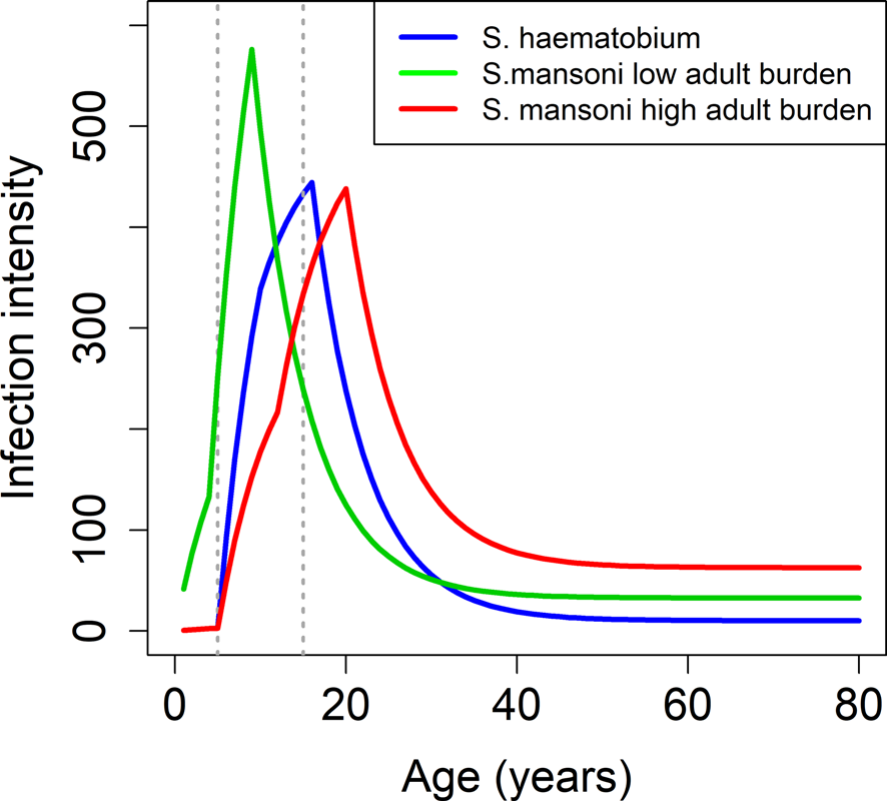
*S. mansoni* and *S. haematobium* age-intensity profiles of infection (eggs/10 mL for *S. haematobium* and eggs/gram for *S. mansoni*, showing low and high burden of adult infection settings).^17,33^

**Figure 2.**
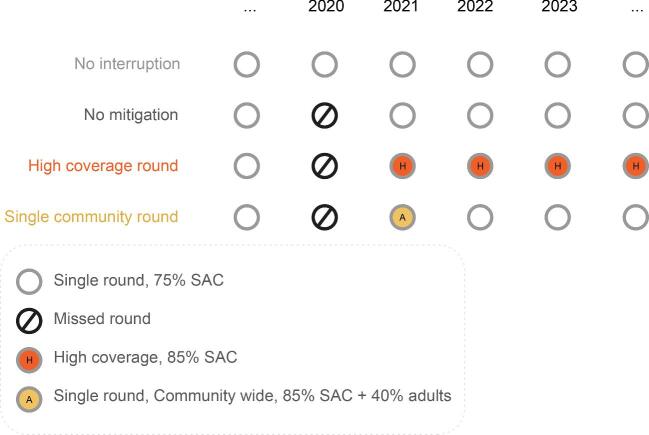
Visual representation of the scenarios and mitigation strategies analysed.

In our investigation, we considered moderate (10–50% baseline prevalence among SAC) and high (50–75% baseline prevalence among SAC) prevalence settings prior to MDA.^2,11^ In addition to this, for *S. mansoni* we used two different age-intensity profiles (low and high adult burden of infection) to determine whether this would differentially influence the impact of missing MDA. We varied the age-intensity profile, as adults can harbour a low to high burden of infection corresponding to their exposure to infection relative to SAC (Figure 1).^17^ In the model, we implemented MDA annually at a 75% coverage level of SAC only.^11^ We simulated one MDA round being missed either early or late (second or sixth round of MDA, respectively) into the programme. For all our scenarios, we determined the time taken to achieve EPHP. After a missed round of MDA, we considered three mitigation strategies (Figure 2): return with annual 75% coverage level of SAC only, return with annual 85% coverage level of SAC only and return with 1 year community-wide coverage (85% SAC+40% adults) followed by 75% coverage of SAC only in the years following. The mitigation strategies and time and length of postponement explored in this article were decided via discussions with the WHO, the Bill and Melinda Gates Foundation and the Neglected Tropical Diseases Modelling Consortium schistosomiasis teams.

For each transmission setting and age profile (Figure 1), we simulated the impact of the different control strategies over a period of 15 y. At each point in time we determined the prevalence of heavy-intensity infections (eggs/g ≥400 for *S. mansoni* and ≥50 eggs/10 mL of urine for *S. haematobium*) in SAC to investigate whether the EPHP goal was achieved.

## Results

We present results for the effect of MDA postponement due to COVID-19 and the impact of mitigation strategies to get back on track towards achieving EPHP by 2030. The results are presented for *S. mansoni* and *S. haematobium* by considering the scenarios and mitigation strategies described in Figure 2.

### Results for *Schistosoma mansoni*

For moderate-transmission settings with a low or high adult burden of infection, missing the second round of MDA (refer to Table 1) requires an additional year of intervention to achieve EPHP, regardless of the mitigation strategy. It should be noted that in lower moderate-transmission settings (i.e. just above 10% SAC prevalence), with 75% coverage the EPHP goal is achieved after one round of MDA, so there is no delay towards the goal when the second MDA is missed. Missing the sixth round of MDA does not have any impact on the time required to achieve the EPHP goal, as the goal has already been reached prior to the sixth round (refer to Table 2).

**Table 1.** Years of MDA to achieve EPHP (≤1% heavy-intensity prevalence in SAC) for *S. mansoni*. The second round of MDA is missed.

Prevalence in SAC	Moderate (10–50%)	High (≥50%)
Time to EPHP if no postponement to annual 75% SAC MDA	Low adult burden: 1–3 y	Low adult burden: 3–8 y
	High adult burden: 1–3 y	High adult burden: 3–NA y
Delay to EPHP if second MDA is missed+return with 75% SAC	Low adult burden: 0–1 y	Low adult burden: 1–2 y
	High adult burden: 0–1 y	High adult burden: 1–NA y
Delay to EPHP if second MDA is missed+return with 85% SAC	Low adult burden: 0–1 y	Low adult burden: 1–0 y
	High adult burden: 0–1 y	High adult burden: 1–NA y
Delay to EPHP if second MDA is missed+return with one community-wide MDA (85% SAC+40% adults) followed by 75% SAC	Low adult burden: 0–1 y	Low adult burden: 1–1 y
	High adult burden: 0–1 y	High adult burden: 1–NA y

NA: not achievable by 2030 (for baseline >59% in SAC). Results are shown for low and high adult burden of infection. For low adult burden and moderate-transmission settings we used R_0_ values of 1.22–1.196 and k values of 0.04–0.24. For high adult burden and moderate-transmission settings we used R_0_ values of 1.245–1.23 and k values of 0.04–0.24. For low adult burden and high-transmission settings we used R_0_ values of 1.198–3.0 and a k value of 0.24. For high adult burden and high-transmission settings we used R_0_ values of 1.24–4 and a k value of 0.24

**Table 2.** Years of MDA to achieve EPHP (≤1% heavy-intensity prevalence in SAC) for *S. mansoni*. The sixth round of MDA is missed.

Prevalence in SAC	Moderate (10–50%)	High (≥50%)
Time to EPHP if no postponement to annual 75% SAC MDA	Low adult burden: 1–3 y	Low adult burden: 3–8 y
	High adult burden: 1–3 y	High adult burden: 3–NA y
Delay to EPHP if sixth MDA is missed+return with 75% SAC	Low adult burden: 0 y	Low adult burden: 0–2 y
	High adult burden: 0 y	High adult burden: 0–NA y
Delay to EPHP if sixth MDA is missed+return with 85% SAC	Low adult burden: 0 y	Low adult burden: 0–2 y
	High adult burden: 0 y	High adult burden: 0–NA y
Delay to EPHP if sixth MDA is missed+return with one community-wide MDA (85% SAC+40% adults) followed by 75% SAC	Low adult burden: 0 y	Low adult burden: 0–2 y
	High adult burden: 0 y	High adult burden: 0–NA y

NA: not achievable by 2030. Results are shown for low and high adult burden of infection settings. For low adult burden and moderate-transmission settings we used R_0_ values of 1.22–1.196 and k values of 0.04–0.24. For high adult burden and moderate-transmission settings we used R_0_ values of 1.245–1.23 and k values of 0.04–0.24. For low adult burden and -transmission settings we used R_0_ values of 1.198–3.0 and a k value of 0.24. For high adult burden and high-transmission settings we used R_0_ values of 1.24–4 and a k value of 0.24.

**Figure 3.**
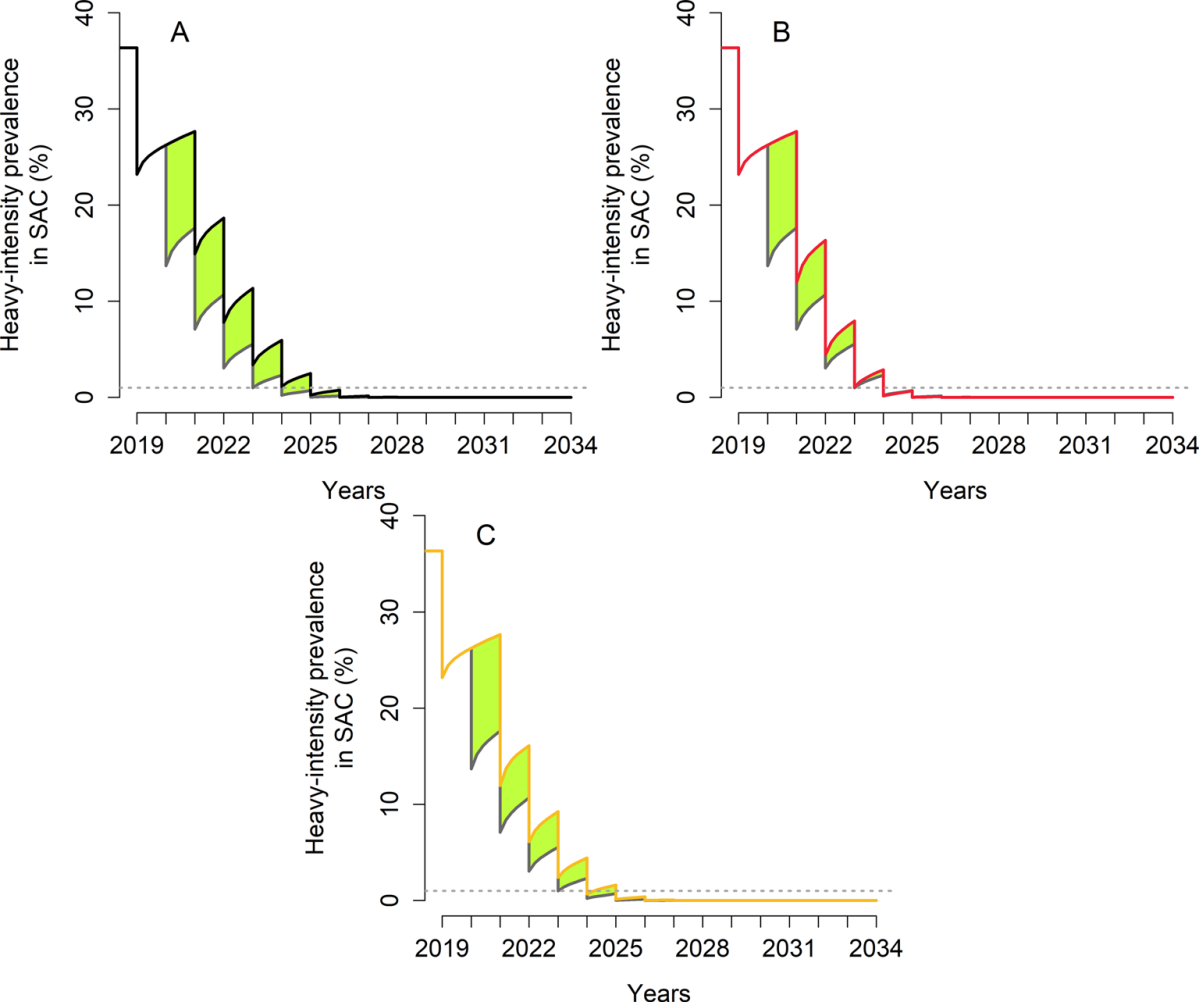
Heavy-intensity prevalence in SAC for *S. mansoni* in high-transmission settings with a low adult burden of infection. The second round of MDA is missed. The grey line shows the prevalence of heavy infection if the treatment had gone ahead as planned. (A) The programme is restarted by treating 75% of SAC (black line). (B) The programme is restarted by treating 85% of SAC (red line). (C) The programme is restarted with one community-wide MDA (85% SAC+40% adults) followed by 75% SAC (yellow line). The green area shows the increased level of infection in the community.

For high-transmission settings with a low adult burden of infection, if the programme is reintroduced at the previous 75% SAC-only coverage, then it is predicted that up to 2 y of delay will result in reaching EPHP (Tables 1 and 2 and Figure 3), regardless of the time MDA is missed. Increasing the coverage level to 85% of SAC or having one round of community-wide MDA requires up to 1 additional year if the second round of MDA is missed. From Figure 3, during the postponement of MDA there is an increase in heavy-intensity infections (illustrated by the black, red and yellow lines). As a result, there is an increase in morbidity, illustrated by the green area. Hence this is an additional burden of infection that would not have happened if the treatment programme had gone as planned.

**Figure 4.**
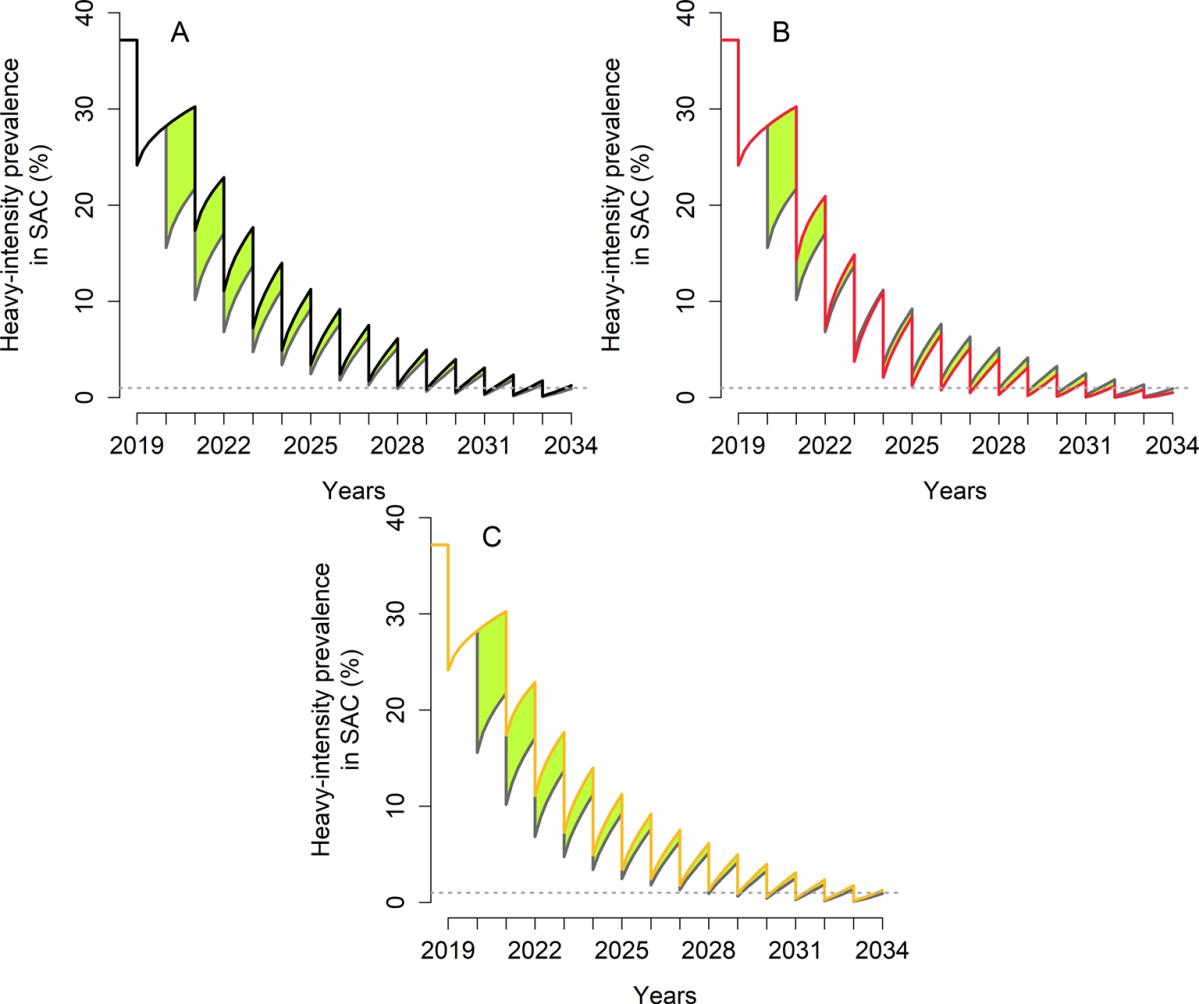
Heavy-intensity prevalence in SAC for *S. mansoni* in high-transmission settings with a high adult burden of infection. The second round of MDA is missed. The grey line shows the prevalence of heavy infection if the treatment had gone ahead as planned. (A) The programme is restarted by treating 75% of SAC (black line). (B) The programme is restarted by treating 85% of SAC (red line). (C) The programme is restarted with one community-wide MDA (85% SAC+40% adults) followed by 75% SAC (yellow line). (D) The programme is restarted by treating 85% of SAC and 40% of adults (blue line). The green area shows the increased level of infection in the community.

For high-transmission settings with a high adult burden, the outcome depends on the baseline SAC prevalence. For a baseline SAC prevalence ≤59% and missing the second round of MDA, a 1-y delay in achieving EPHP is predicted (Supplementary Figure S1). This holds for any mitigation strategy considered. However, for a baseline SAC prevalence >59%, EPHP is not achieved by 2030 regardless of the mitigation strategy described in Figure 2 (refer to Tables 1 and 2 and Figure 4). This is because MDA of SAC only has a small impact on reducing transmission. To achieve EPHP within a shorter time frame, higher coverage of SAC and treating adults would be needed for this setting. These coverage levels can be determined by collecting SAC and adult data once programmes resume.^17^ In Supplementary Figure S2, it is shown that once the MDA programme resumes, increasing the SAC coverage to 85% and including 40% of adults for every MDA round can achieve the goal by 2030.

If the sixth round of MDA is missed and the baseline SAC prevalence is ≤59%, no additional year of intervention is required, regardless of the mitigation strategy (Table 2). However, for baseline SAC prevalence above this threshold, EPHP is not achieved by 2030 with any of the mitigation strategies considered in Figure 2 (see Supplementary Figure S3). Similarly, as when the second round of MDA is postponed, increasing the SAC coverage to 85% and treating 40% of adults in every round after the programme resumes can achieve the EPHP goal by 2030.

Our simulations show that missing the second round of MDA for a baseline SAC prevalence of 30% (moderate transmission setting) may take from 4 to 10 y for SAC prevalence to catch up to the state where no MDA rounds are missed (depending on the scenario and adult burden of infection; refer to Supplementary Tables S3 and S4). Missing the sixth round of MDA does not have any impact on the time required to achieve the EPHP goal, but it might take up to 5 y for the SAC prevalence to catch up to what would have been achieved without missing MDA rounds.

For a baseline SAC prevalence of 70% (high transmission setting) with a low adult burden, it may take from 5 to 12 y for the SAC prevalence to catch up (Supplementary Table S3). For the high-transmission setting with a high adult burden, it is predicted that it may take >3 y for the SAC prevalence to get back to the level with no missed rounds (depending on the scenario; refer to Supplementary Table S4).

### Results for *S. haematobium*

For moderate-transmission settings with no postponement of MDA, it takes up to 2 y for EPHP to be achieved (Tables 3 and 4). For lower moderate-prevalence settings (i.e. just above 10% SAC prevalence), the heavy-intensity prevalence in SAC may be <1% before the start of treatment. Therefore the EPHP goal is met without any MDA intervention. Missing the second round of MDA (Table 3) will require up to 1 additional year to achieve the goal, regardless of the mitigation strategy. However, missing the sixth round of MDA does not have any effect on the goal because it was achieved prior to the missed MDA (Table 4).

**Table 3.** Years of MDA to achieve EPHP (≤1% heavy-intensity prevalence in SAC) for *S. haematobium*. The second round of MDA is missed.

Prevalence in SAC	Moderate (10–50%)	High (≥50%)
Time to EPHP if no postponement to annual 75% SAC MDA	0–2 y	2–9 y
Delay to EPHP if second MDA is missed+return with 75% SAC	0–1 y	1 y
Delay to EPHP if second MDA is missed+return with 85% SAC	0–1 y	1–0 y
Delay to EPHP if second MDA is missed+return with one community-wide MDA (85% SAC + 40% adults) followed by 75% SAC	0–1 y	1–0 y

For moderate-transmission settings we used R_0_ values of 1.203–1.184 and k values of 0.04–0.24. For high-transmission settings we used R_0_ values of 1.185–3.0 and a k value of 0.24.

**Table 4.** Years of MDA to achieve EPHP (≤1% heavy-intensity prevalence in SAC) for *S. haematobium*. The sixth round of MDA is missed.

Prevalence in SAC	Moderate (10–50%)	High (≥50%)
Time to EPHP if no postponement to annual 75% SAC MDA	0–2 y	2–9 y
Delay to EPHP if sixth MDA is missed+return with 75% SAC	0 y	0–1 y
Delay to EPHP if sixth MDA is missed+return with 85% SAC	0 y	0 y
Delay to EPHP if sixth MDA is missed+return with one community-wide MDA (85% SAC+40% adults) followed by 75% SAC	0 y	0–1 y

For moderate-transmission settings we used R_0_ values of 1.203–1.184 and k values of 0.04–0.24. For high-transmission settings we used R_0_ values of 1.185–3.0 and a k value of 0.24.

**Figure 5.**
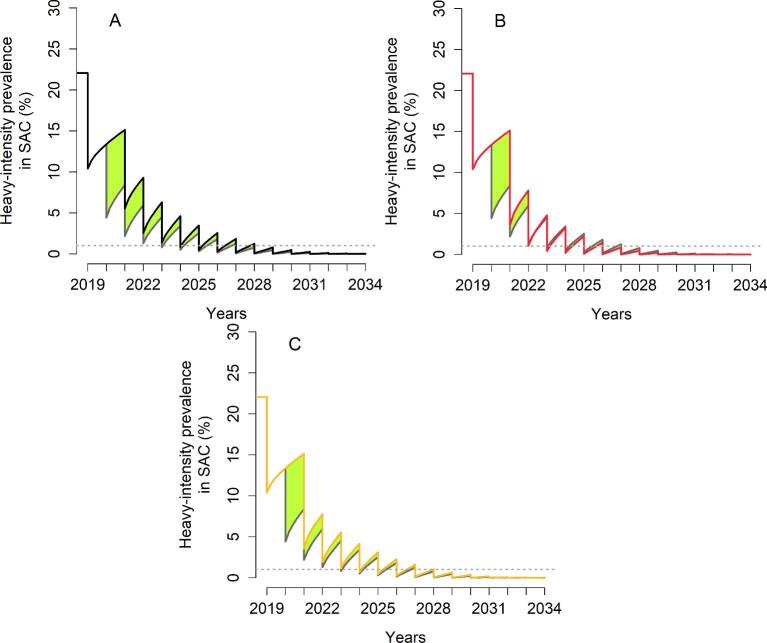
Heavy-intensity prevalence in SAC for *S. haematobium*. The second round of MDA is missed. The grey line gives the prevalence of heavy infection if the treatment had gone ahead as planned. (A) The programme is restarted by treating 75% of SAC (black line). (B) The programme is restarted by treating 85% of SAC (red line). (C) The programme is restarted with one community-wide MDA (85% SAC+40% adults) followed by 75% SAC (yellow line). The green area shows the increased level of infection in the community.

For high-transmission settings, depending on the baseline SAC prevalence, it takes 2–9 y to achieve EPHP (no delay in MDA treatment). For scenarios where it takes 2 y to EPHP, missing the second round of MDA will require 1 additional year of intervention, regardless of the mitigation strategies. For scenarios where it takes >2 y to achieve EPHP, a 1 y delay is also expected when the programme is reintroduced at the previous coverage level after missing the second round of MDA (refer to Table 3 and Figure 5). However, increasing the coverage level to 85% SAC (or having one round of community-wide treatment) does not require the additional year of MDA. Missing the sixth round of MDA has a smaller effect on the time to achieve the goal. Increasing the coverage level to 85% SAC only does not require any additional years of treatment (Table 4).

## Discussion

We have presented analyses of the impact of delaying MDA due to COVID-19 and considered various mitigation strategies to get the programme back on track for achieving EPHP by 2030 for both *S. mansoni* and *S. haematobium*. We assumed that MDA would be delayed for 1 y, either early or late into the programme (second or sixth round of treatment). Once the programme resumes, the delay in achieving EPHP is calculated for various mitigation strategies.

For *S. mansoni*, our analyses suggest that postponing MDA for 1 y can delay the EPHP goal by up to 2 y, with the greatest impact being in high-transmission settings. This is due to the fact that in these settings the resurgence of infection is greater and consequently the number of rounds required to catch up will also be greater. A whole-community MDA round or an increase in SAC coverage (from 75% to 85%) after programmes restart could help accelerate progress towards EPHP by reducing the delay to target by up to 1 y.

High-transmission settings with a high adult burden of infection might not achieve the EPHP goal, regardless of the postponement.^24,26^ For these settings, an increase in SAC coverage and inclusion of adults is necessary to achieve EPHP by 2030. We acknowledge that due to limited praziquantel supplies (donations),^27^ including adults in treatment may not be feasible in all areas. Hence it is important that surveys are conducted to collect SAC and adult data to determine the optimal coverage levels and whether adult treatment is required.^24^ This will then allow for community-wide treatment to be prioritised as necessary in high-transmission settings where there is a high adult burden of infection.

For *S. haematobium*, postponing MDA for 1 y can delay the EPHP goal by up to 1 y. Annual 75% SAC-only treatment is sufficient for achieving EPHP by 2030, even when MDA is postponed for 1 y.

For both *S. mansoni* and *S. haematobium*, missing MDA further into the programme has a lower impact on achieving the EPHP goal than postponing MDA early in the programme. This is because the goal is achieved before the delay occurs and hence it takes longer to return to pre-MDA levels. Additionally, we find that if the EPHP goal is achieved before postponement of the MDA programme, the goal can remain unaffected by the postponement. Using intensive mitigation strategies (as described in Figure 2) can increase the probability of achieving the target.

Overall, postponing MDA for 1 y results in a delay of up to 2 y for achieving EPHP. The impact of missing MDA depends on the baseline prevalence prior to treatment, the burden of infection in adults and the time at which we miss MDA (early or late into the programme).

However, care should be taken when deciding to stop MDA after EPHP has been achieved, as there will be a risk of resurgence/bounce back. This is because the overall prevalence might still be high, so infection persists despite the heavy-intensity prevalence in SAC being reduced to ≤1%.^28^ Additionally, it is predicted that it takes longer for the SAC prevalence to catch up to what would have been achieved by full MDA rounds than it takes for the heavy-intensity prevalence (Supplementary Tables S3 and S4).

In this study we assumed that control programmes will return to their pre-COVID-19 effectiveness within 1 y, but this might not be feasible for various reasons. Training programmes may have been disrupted by COVID-19 and health workers might have been redeployed to other tasks. Another important factor is that schools may not open when the programme restarts or parents may decide not to send their children back to school. As the MDA programme is mainly focused on SAC, this will have a major impact on the mitigation strategies. We also need to take into consideration the fact that stocks of praziquantel in government warehouses may exceed their expiry dates during the delay or that praziquantel production and supply chains of MDA treatment may be disrupted due to travel restrictions. Thus it might take some time to achieve the desired coverage once the programme restarts. As a result, postponing programmes for longer or returning with reduced effectiveness will mean that we might ultimately be facing longer delays in achieving the EPHP goal.

When considering a longer postponement of MDA, e.g. 18 months, analyses suggest that the EPHP goal could be delayed by an extra 6 months, depending on the transmission setting and adult burden of infection (Supplementary Tables S5 and S6). Hence the longer the delay, the longer it will take programmes to achieve EPHP. Mitigation strategies upon resumption will be increasingly important in areas where programmes are delayed longer.

It is important to note one important caveat on the predictions made in this study. It was assumed that for a fixed MDA coverage level, treatment is done at random in the population. This may not be the case, as persistent non-adherers to treatment (due to many different factors) are an important feature of most MDA-based control programmes. If this is the case for treating schistosome infections, our predictions may err on the side of being too optimistic, as persistent non-adherers can harbour worms, creating a reservoir of untreated infection. Previous mathematical modelling for schistosomiasis has shown that individual compliance to treatment has a great impact on the probability of elimination.^22^ Random compliance could achieve the elimination of schistosomiasis with a high probability, whereas semi-systematic treatment reduced the probability of elimination by half and systematic compliance reduced this probability to zero even after many rounds of MDA.^22^ This clearly shows how important individual compliance to treatment is in determining the impact of MDA in achieving morbidity control and elimination as opposed to just recording the MDA coverage, as commonly happens. If we improve compliance to treatment, we also need to ensure coverage remains high. This issue can be addressed if data on individual compliance are recorded, but very little attention has been paid to this in the monitoring and evaluation of schistosomiasis control programmes.

It should also be noted that in this study we have not included the impact of acquired immunity in achieving the EPHP. With our current knowledge, it is not possible to infer immunity parameters such as strength and duration. However, a degree of immunity is believed to slowly build up over long periods of exposure, which can lessen the impact of MDA in achieving morbidity control and elimination, as repeated rounds of MDA can reduce the level of acquired immunity over time.^29,30^ This means that the average worm burden in adults will be increased to pre-MDA levels. Similarly, human population movement can affect the impact of MDA programmes by lowering the probability of elimination.^31^

In this study we used an age-structured deterministic model, but its analogue individual-based stochastic model can be employed if interest lies in the exact probability of achieving the target as opposed to a yes/no outcome.^23,8^ However, the mean derived from the stochastic model is identical to the deterministic model prediction.

The model-based predictions can be tested once the MDA programme is resumed, as we expect to see a large increase in the prevalence of infection after a long period of no intervention, particularly in high-transmission settings. Data collection on SAC and adults needs to be done at the start of the resumed intervention. The ongoing Geshiyaro Project can address this.^32^

## Conclusions

In this study we show that postponement of rounds of MDA due to the COVID-19 pandemic will lead to an increase in *S. mansoni* and *S. haematobium* infection. As a result, more resources will be needed to reach the 2030 goal of EPHP once the MDA programmes restart. The transmission setting, duration of the delay in delivering MDA, stage of the programme and age-intensity profile will all have an impact on achieving WHO goals for control of both morbidity and transmission. Mitigation strategies can help in accelerating progress towards EPHP by 2030. In some high-transmission settings, EPHP may not be reached regardless of the length of the delay and hence, upon resumption, it is important that surveys are done to collect SAC and adult infection data in order to determine the desired coverage levels for MDA to reach the defined control objectives. We hope this study will provide health workers with important quantitative tools to assess what mitigation strategies are best applied in given epidemiological settings.

## Supplementary Material

traa202_Supplemental_File

## Data Availability

The simulations results of this article will be shared upon reasonable request to the corresponding author.
