# Supplementary material for: Disruptions to schistosomiasis programmes due to COVID-19: an analysis of potential impact and mitigation strategies
Source: Trans R Soc Trop Med Hyg. 2021 Jan 29;115(3):236–44. doi: 10.1093/trstmh/traa202 (PMC7928593; doi:10.1093/trstmh/traa202)
Supplement: traa202_Supplemental_File [file traa202_supplemental_file.docx]

**Table S1:** Parameter values used for *Schistosoma mansoni.*

| **Parameter** | **Value** | **Source** |
| --- | --- | --- |
| Fecundity | 0.34 eggs/female/sample | ^1–3^ |
| Egg distribution within the individual | 0.87 | ^1,3^ |
| Aggregation parameter | 0.24 | ^4–8^ |
| Density dependent fecundity | 0.0007/female worm | ^9,10^ |
| Worm lifespan | 5.7 years | ^1,4,11^ |
| Drug efficacy | 86.3% | ^4,12^ |
| Low adult burden setting: Age specific contact rates for 0-4, 5-9, 10-15, 16+ years old | 0.01, 1.2, 1, 0.02 | ^10,13^ |
| High adult burden setting: Age specific contact rates for 0-4, 5-11, 12-19, 20+ years old | 0.01, 0.61, 1, 0.12 | ^10,13^ |
| Prevalence of infection | Percentage of population having > 0 eggs per gram [epg] | - |
| Heavy-intensity infection prevalence | Percentage of population having ≥ 400 epg | ^14,15^ |
| Human demography | Based on Uganda’s demographic profile | ^16,17^ |

**Table S2:** Parameter values used for *S. haematobium*.

| **Parameter** | **Value** | **Source** |
| --- | --- | --- |
| Fecundity | 0.3 | ^4^ |
| Egg distribution within the individual | 0.5 | ^4^ |
| Aggregation parameter | 0.24 | ^4–8^ |
| Density dependent fecundity | 0.0006/female worm | ^4^ |
| Worm lifespan | 4 years | ^1,4,11^ |
| Drug efficacy | 94% | ^4,12^ |
| Age specific contact rates for 0-4, 5-9, 10+ years old | 0.3, 1, 0.02 | ^4^ |
| Prevalence of infection | Percentage of population having > 0 eggs/10ml | - |
| Heavy-intensity infection prevalence | Percentage of population having ≥ 50 eggs/10ml | ^14,15^ |
| Human demography | Based on Uganda’s demographic profile | ^16,17^ |

**Table S3: Low adult burden of infection for *S. mansoni*.** Years of MDA required for the SAC prevalence to catch-up after the programme is resumed (the second or the sixth round of MDA is missed for one year).

| **Prevalence in SAC prior to treatment** | **Annual 75% SAC MDA is resumed** | **Annual 85% SAC MDA is resumed** | **One round of community-wide MDA is delivered, before returning to annual 75% SAC MDA** |
| --- | --- | --- | --- |
| Moderate (10-50%)    Baseline prevalence in SAC: 30% | **Miss the 2^nd^ round:** SAC prevalence catches up after 6 years.  **Miss the 6^th^ round:** SAC prevalence catches up after 3 years. | **Miss the 2^nd^ round:** SAC prevalence catches up after 5 years.  **Miss the 6^th^ round:** SAC prevalence catches up after 2 years. | **Miss the 2^nd^ round:** SAC prevalence catches up after 6 years.  **Miss the 6^th^ round:** SAC prevalence catches up after 2 years. |
| High (≥50%)    Baseline prevalence in SAC: 70% | **Miss the 2^nd^ round:** SAC prevalence catches up after 12 years.  **Miss the 6^th^ round:** SAC prevalence catches up after 8 years. | **Miss the 2^nd^ round:** SAC prevalence catches up after 5 years.  **Miss the 6^th^ round:** SAC prevalence catches up after 5 years. | **Miss the 2^nd^ round:** SAC prevalence catches up after 9 years.  **Miss the 6^th^ round:** SAC prevalence catches up after 6 years. |

**Table S4: High adult burden of infection for *S. mansoni*.** Years of MDA required for the SAC prevalence to catch-up after the programme is resumed (the second or the sixth round of MDA is missed for one year).

| **Prevalence in SAC prior to treatment** | **Annual 75% SAC MDA is resumed** | **Annual 85% SAC MDA is resumed** | **One round of community-wide MDA is delivered, before returning to annual 75% SAC MDA** |
| --- | --- | --- | --- |
| Moderate (10-50%)    Baseline prevalence in SAC: 30% | **Miss the 2^nd^ round:** SAC prevalence catches up after 10 years.  **Miss the 6^th^ round:** SAC prevalence catches up after 5 years. | **Miss the 2^nd^ round:** SAC prevalence catches up after 4 years.  **Miss the 6^th^ round:** SAC prevalence catches up after 3 years. | **Miss the 2^nd^ round:** SAC prevalence catches up after 4 years.  **Miss the 6^th^ round:** SAC prevalence catches up after 3 years. |
| High (≥50%)    Baseline prevalence in SAC: 70% | **Miss the 2^nd^ round:** SAC prevalence does not catch up  **Miss the 6^th^ round:** SAC prevalence does not catch up. | **Miss the 2^nd^ round:** SAC prevalence catches up after 3 years.  **Miss the 6^th^ round:** SAC prevalence catches up after 3 years. | **Miss the 2^nd^ round:** SAC prevalence catches up after 5 years.  **Miss the 6^th^ round:** SAC prevalence catches up after 3 years. |

In **Table S5** and **Table S6**, we show the impact of postponing MDA for 18 months in achieving the EPHP goal.

**Table S5:** The impact of missing the second round of MDA in achieving the elimination as a public health problem goal (≤1% heavy-intensity prevalence in SAC) and suggestions for programmatic adaptations when the EPHP goal is not achieved for Schistosoma mansoni. Results are shown for low and high adult burden of infection settings.

| **Prevalence in SAC** | **EPHP reached until 2030 with no interruption** (annual 75% SAC MDA delivered as planned)? | **EPHP reached until 2030 with no mitigation** (annual 75% SAC MDA is resumed)? | **Programatic adaptations** |
| --- | --- | --- | --- |
| **Moderate** (10-50%) | Low adult burden: Yes within 1-3 years  High adult burden: Yes, within 1–3 years | Low adult burden: Yes within 1-3 years  High adult burden: Yes within 1-5.5 years | Not required |
| **High** (≥50%) | Low adult burden: Yes within 3-8 years  High adult burden: Within 3-7 years for baseline less than 59%. Not achieved for baseline higher than 59% in SAC | Low adult burden: yes, within 3-9.5 years.  High adult burden: Within 5.5-9 years for baseline less than 59%. Not achieved for baseline higher than 59% in SAC | For high adult burden of infection and baseline in SAC over 59% do community wide treatment (85% SAC +40% adults) to achieve EPHP within 10.5 years. |

**Table S6:** The impact of missing the sixth round of MDA in achieving the elimination as a public health problem goal (≤1% heavy-intensity prevalence in SAC) and suggestions for programmatic adaptations when the EPHP goal is not achieved for Schistosoma mansoni. Results are shown for low and high adult burden of infection settings.

| **Prevalence in SAC** | **EPHP reached until 2030 with no interruption (**annual 75% SAC MDA delivered as planned)? | **EPHP reached until 2030 with no mitigation** (annual 75% SAC MDA is resumed)? | **Programatic adaptations^*^** |
| --- | --- | --- | --- |
| **Moderate** (10-50%) | Low adult burden: Yes within 1-3 years  High adult burden: Yes, within 1–3 years | Low adult burden: Yes within 1-3 years  High adult burden: Yes within 1-3 years | Not required |
| **High** (≥50%) | Low adult burden: Yes within 3-8 years  High adult burden: Within 3-7 years for baseline less than 59%. Not achieved for baseline higher than 59% in SAC | Low adult burden: yes, within 3-10.5 years.  High adult burden: Within 3-7 years for baseline less than 59%. Not achieved for baseline higher than 59% in SAC | For high adult burden of infection and baseline in SAC over 59% do community wide treatment (85% SAC +40% adults) to achieve EPHP within 13.5 years. |

To increase the modelling impact to policy and decision makers, we summarize in **Table S7** the five modelling principles, as described in, ^18^ and how they are achieved in the manuscript.

**Table S7:** The Policy-Relevant Items for Reporting Models in Epidemiology of Neglected Tropical Diseases (PRIME-NTD) .^18^

| **Principle** | **What has been done to satisfy the principle?** | **Where in the manuscript is this described?** |
| --- | --- | --- |
| **Stakeholder engagement** | Work has been presented at the following WHO webinars: (i) Neglected Tropical Diseases and COVID-19: Impact on Programme Implementation; and (ii) A Research Agenda for NTD Programmes Affected by the COVID-19 Pandemic. | - |
| **Complete model documentation** | Transmission model and mitigation strategies are described in the manuscript. | Methods section (Transmission model)^19,20^ |
| **Complete description of data used** | Data and parameters used are described in the manuscript. | Figure 1 and Table S1 |
| **Communicating uncertainty** | We have considered two age-intensity profiles for *S. mansoni* and different stages of the programme for MDA interruption. | Methods and Results sections |
| **Testable model outcomes** | The model outcomes can be tested by the ongoing Geshiyaro project and by collecting data once programmes resume. | Discussion section |


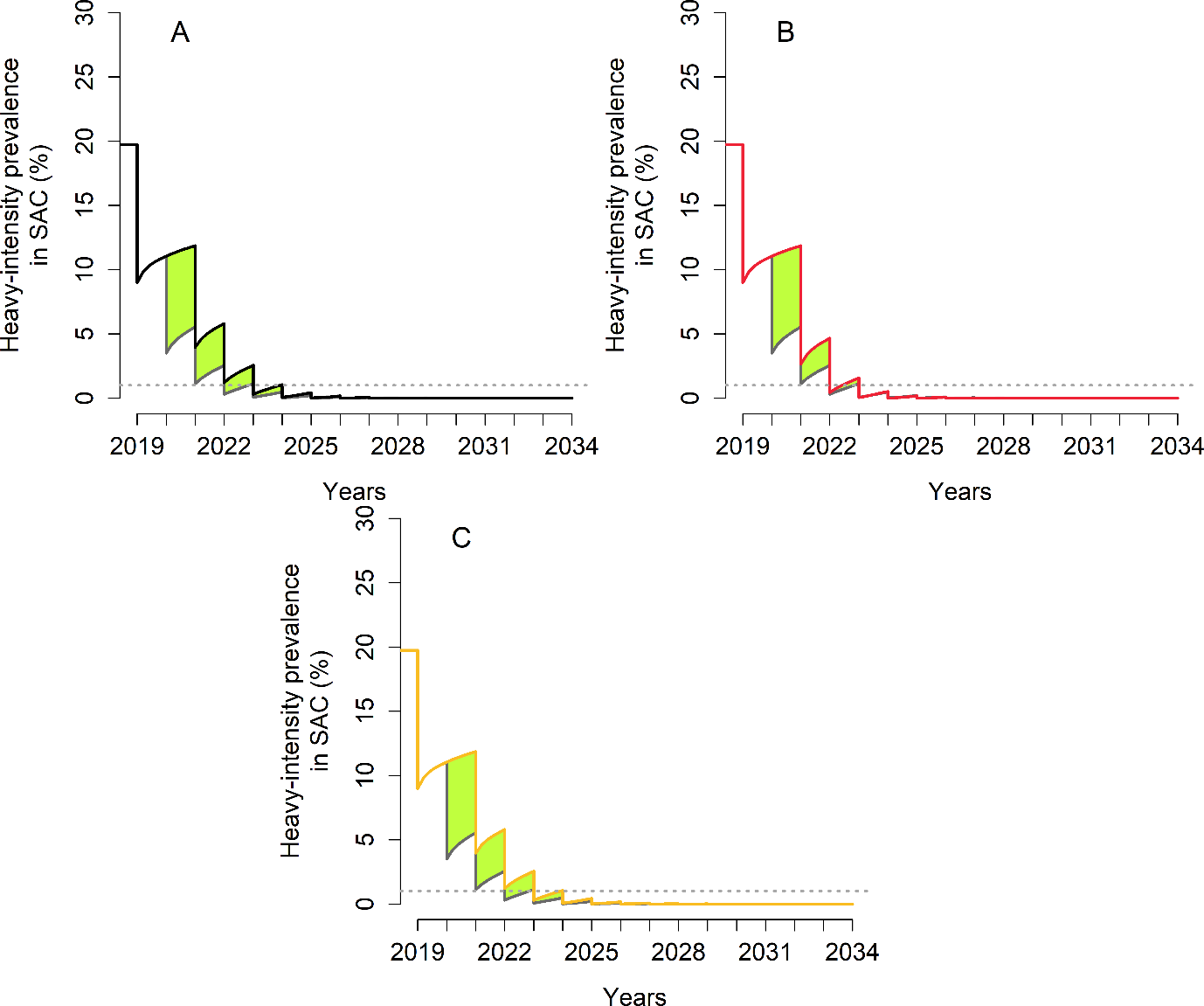


**Figure S1:** Heavy-intensity prevalence in SAC for *S. mansoni* in high transmission settings (baseline prevalence in SAC=59%) with a high adult burden of infection. The second round of MDA is missed. The grey line gives the prevalence of heavy infection if the treatment had gone ahead as planned. (**A**) the programme is restarted by treating 75% of SAC (black line). (**B**) the programme is restarted by treating 85% of SAC (red line). (**C**) the programme is restarted with one community-wide MDA (85% SAC + 40% adults) followed by 75% SAC (yellow line). The green area shows the increased level of infection in the community.


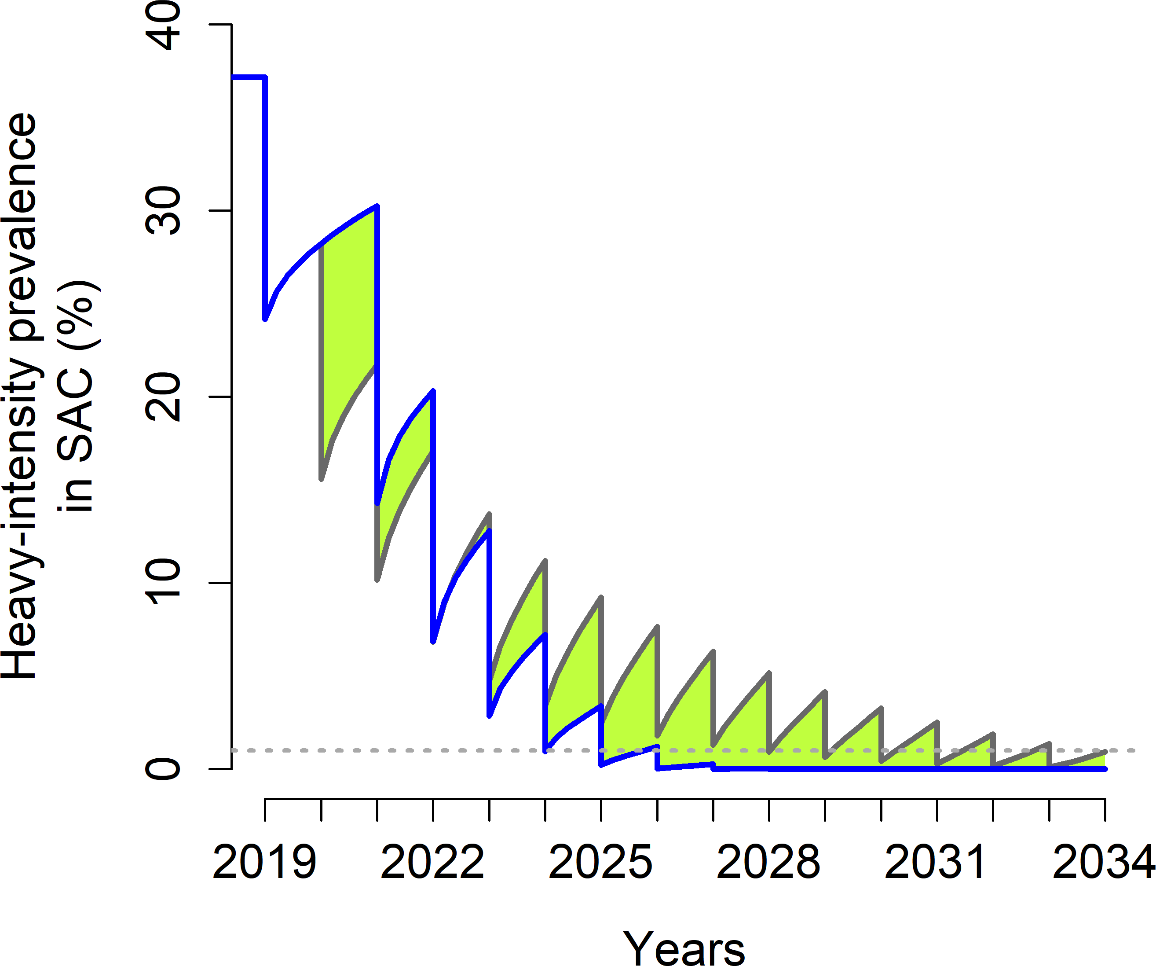


**Figure S2:** Heavy-intensity prevalence in SAC for *S. mansoni* in high transmission settings with a high adult burden of infection. The second round of MDA is missed. The grey line gives the prevalence of heavy infection if the treatment had gone ahead as planned. The blue line gives the prevalence of heavy infection if the programme is restarted by treating 85% of SAC and 40% of adults. The green area shows the increased level of infection in the community.


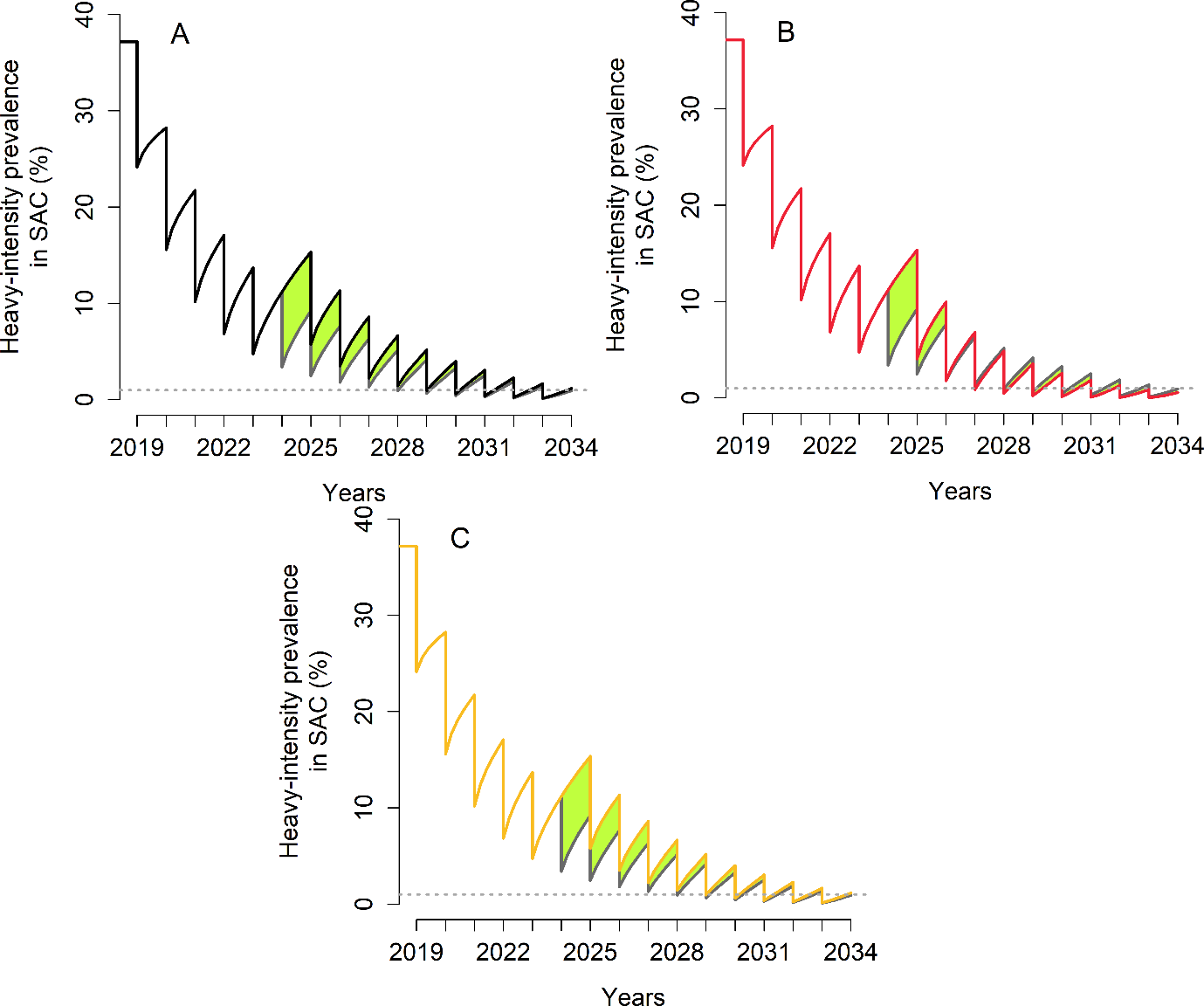


**Figure S3:** Heavy-intensity prevalence in SAC for *S. mansoni* in high transmission settings with a high adult burden of infection. The sixth round of MDA is missed. The grey line gives the prevalence of heavy infection if the treatment had gone ahead as planned. (**A**) the programme is restarted by treating 75% of SAC (black line). (**B**) the programme is restarted by treating 85% of SAC (red line). (**C**) the programme is restarted with one community-wide MDA (85% SAC + 40% adults) followed by 75% SAC (yellow line). The green area shows the increased level of infection in the community.

**References**

1. Anderson RM, May RM. Population dynamics of human helminth infections: Control by chemotherapy. *Nature*. Published online 1982. doi:10.1038/297557a0

2. De Vlas SJ, Gryseels B, Van Oortmarssen GJ, Polderman AM, Habbema JD. A model for variations in single and repeated egg counts in Schistosoma mansoni infections. *Parasitology*. 1992;104 ( Pt 3):451-460. Accessed April 30, 2019. http://www.ncbi.nlm.nih.gov/pubmed/1641245

3. de Vlas S, Nagelkerke N, Habbema J. Statistical models for estimating prevalence and incidence of parasitic diseases. *Stat Methods Med Res*. Published online 1993. doi:10.1177/096228029300200102

4. Truscott JE, Gurarie D, Alsallaq R, Toor J, Yoon N, Farrell SH, Turner HC, Phillips AE, Aurelio HO, Ferro J, King CH, Anderson RM. A comparison of two mathematical models of the impact of mass drug administration on the transmission and control of schistosomiasis. *Epidemics*. 2017;18:29-37. doi:10.1016/j.epidem.2017.02.003

5. Anderson RM, Turner HC, Farrell SH, Truscott JE. Studies of the Transmission Dynamics, Mathematical Model Development and the Control of Schistosome Parasites by Mass Drug Administration in Human Communities. *Adv Parasitol*. Published online 2016. doi:10.1016/bs.apar.2016.06.003

6. Chan MS, Guyatt HL, Bundy DAP, Booth M, Fulford AJC, Medley GF. The development of an age structured model for schistosomiasis transmission dynamics and control and its validation for Schistosoma mansoni. *Epidemiol Infect*. Published online 1995. doi:10.1017/S0950268800058453

7. Kura K, Truscott JE, Toor J, Anderson RM. Modelling the impact of a Schistosoma mansoni vaccine and mass drug administration to achieve morbidity control and transmission elimination. Akullian A, ed. *PLoS Negl Trop Dis*. 2019;13(6):e0007349. doi:10.1371/journal.pntd.0007349

8. Kura K, Collyer BS, Toor J, Truscott JE, Hollingsworth TD, Keeling MJ, Anderson RM. Policy implications of the potential use of a novel vaccine to prevent infection with Schistosoma mansoni with or without mass drug administration. *Vaccine*. 2020;38(28):4379-4386. doi:10.1016/j.vaccine.2020.04.078

9. Vos T, Allen C, Arora M, Barber RM, Brown A, Carter A, Casey DC, Charlson FJ, Chen AZ, Coggeshall M, Cornaby L, Dandona L, Dicker DJ, Dilegge T, Erskine HE, Ferrari AJ, Fitzmaurice C, Fleming T, Forouzanfar MH, Fullman N, Goldberg EM, Graetz N, Haagsma JA, Hay SI, Johnson CO, Kassebaum NJ, Kawashima T, Kemmer L, Khalil IA, Kyu HH, Leung J, Lim SS, Lopez AD, Marczak L, Mokdad AH, Naghavi M, Nguyen G, Nsoesie E, Olsen H, Pigott DM, Pinho C, Rankin Z, Reinig N, Sandar L, Smith A, Stanaway J, Steiner C, Teeple S, Thomas BA, Troeger C, Wagner JA, Wang H, Wanga V, Whiteford HA, Zoeckler L, Alexander LT, Anderson GM, Bell B, Bienhoff K, Biryukov S, Blore J, Brown J, Coates MM, Daoud F, Estep K, Foreman K, Fox J, Friedman J, Frostad J, Godwin WW, Hancock J, Huynh C, Iannarone M, Kim P, Kutz M, Masiye F, Millear A, Mirarefin M, Mooney MD, Moradi-Lakeh M, Mullany E, Mumford JE, Ng M, Rao P, Reitsma MB, Reynolds A, Roth GA, Shackelford KA, Sivonda A, Sligar A, Sorensen RJD, Sur P, Vollset SE, Woodbrook R, Zhou M, Murray CJL, Ellenbogen RG, Kotsakis GA, et al. Global, regional, and national incidence, prevalence, and years lived with disability for 310 diseases and injuries, 1990–2015: a systematic analysis for the Global Burden of Disease Study 2015. *Lancet*. Published online 2016. doi:10.1016/S0140-6736(16)31678-6

10. Turner HC, Truscott JE, Bettis AA, Farrell SH, Deol AK, Whitton JM, Fleming FM, Anderson RM. Evaluating the variation in the projected benefit of community-wide mass treatment for schistosomiasis: Implications for future economic evaluations. *Parasites and Vectors*. Published online 2017. doi:10.1186/s13071-017-2141-5

11. Fulford AJ, Butterworth AE, Ouma JH, Sturrock RF. A statistical approach to schistosome population dynamics and estimation of the life-span of Schistosoma mansoni in man. *Parasitology*. 1995;110 ( Pt 3):307-316. Accessed April 30, 2019. http://www.ncbi.nlm.nih.gov/pubmed/7724238

12. Zwang J, Olliaro PL. Clinical Efficacy and Tolerability of Praziquantel for Intestinal and Urinary Schistosomiasis—A Meta-analysis of Comparative and Non-comparative Clinical Trials. Jones MK, ed. *PLoS Negl Trop Dis*. 2014;8(11):e3286. doi:10.1371/journal.pntd.0003286

13. Toor J, Turner HC, Truscott JE, Werkman M, Phillips AE, Alsallaq R, Medley GF, King CH, Anderson RM. The design of schistosomiasis monitoring and evaluation programmes: The importance of collecting adult data to inform treatment strategies for Schistosoma mansoni. Shiff C, ed. *PLoS Negl Trop Dis*. 2018;12(10):e0006717. doi:10.1371/journal.pntd.0006717

14. WHO Expert Committee on the Control of Schistosomiasis. *Prevention and Control of Schistosomiasis and Soil-Transmitted Helminthiasis : Report of a WHO Expert Committee.* World Health Organization; 2002.

15. WHO | Schistosomiasis: progress report 2001–2011, strategic plan 2012–2020. *WHO*. Published online 2017. Accessed April 30, 2019. https://www.who.int/neglected_diseases/resources/9789241503174/en/

16. Anderson R, Truscott J, Hollingsworth TD. The coverage and frequency of mass drug administration required to eliminate persistent transmission of soil-transmitted helminths. *Philos Trans R Soc B Biol Sci*. Published online 2014. doi:10.1098/rstb.2013.0435

17. Pullan RL, Kabatereine NB, Quinnell RJ, Brooker S. Spatial and genetic epidemiology of hookworm in a rural community in Uganda. *PLoS Negl Trop Dis*. Published online 2010. doi:10.1371/journal.pntd.0000713

18. Behrend MR, Basáñez MG, Hamley JID, Porco TC, Stolk WA, Walker M, de Vlas SJ. Modelling for policy: The five principles of the neglected tropical diseases modelling consortium. *PLoS Negl Trop Dis*. Published online 2020. doi:10.1371/journal.pntd.0008033

19. Anderson RM, Turner HC, Farrell SH, Truscott JE. Studies of the Transmission Dynamics, Mathematical Model Development and the Control of Schistosome Parasites by Mass Drug Administration in Human Communities. In: *Advances in Parasitology*. Vol 94. ; 2016:199-246. doi:10.1016/bs.apar.2016.06.003

20. Anderson RM, May RM. Helminth Infections of Humans: Mathematical Models, Population Dynamics, and Control. *Adv Parasitol*. Published online 1985. doi:10.1016/S0065-308X(08)60561-8
